# Development and application of a physiologically-based pharmacokinetic model for ractopamine in goats

**DOI:** 10.3389/fvets.2024.1399043

**Published:** 2024-10-02

**Authors:** Jing Ai, Yunfeng Gao, Fan Yang, Zhen Zhao, Jin Dong, Jing Wang, Shiyi Fu, Ying Ma, Xu Gu

**Affiliations:** ^1^Institute of Feed Research of Chinese Academy of Agricultural Sciences, Beijing, China; ^2^Heilongjiang Technical Appraisal Station of Agricultural Products, Veterinary Pharmaceuticals and Feed, Harbin, China; ^3^College of Animal Science and Technology, Henan University of Science and Technology, Luoyang, China; ^4^Beijing Nutrient Source Research Institute Co., Ltd., Beijing, China; ^5^ZiBo Government Service Center, Zibo, Shandong, China; ^6^Jiangxi Agricultural Technology Extension Center, Nanchang, China

**Keywords:** ractopamine, goats, physiologically-based pharmacokinetic model, physiological parameters, residues

## Abstract

Physiologically Based Pharmacokinetic (PBPK) models can provide forecasts of the drug residues within the organism. Ractopamine (RAC) is a typical β-agonist. In this study, we developed a PBPK model for RAC in goats. The goal was to predict the distribution of the drug after multiple oral administrations. The preliminary PBPK model for RAC in goats performed well in predicting the drug’s distribution in most tissues. In our sensitivity analysis, we found that the parameter of Qclu (Blood Flow Volume through Lungs) had the greatest impact on the RAC concentrations in plasma, liver, and kidney and was the most sensitive parameter. Furthermore, our study aimed to assess the withdrawal time (WT) of RAC in different tissues after RAC long-term exposure in goats. We found that the WT of RAC in the kidney was the longest, lasting for 13  days. Overall, the insights gained from this study have important implications for optimizing drug administration in goats and ensuring appropriate withdrawal times to prevent any potential risks.

## 1 Introduction

Ractopamine (RAC) is a phenolamine *β*-adrenoceptor agonist commonly used in animal production as a second-generation clenbuterol. *β*-agonists are known for their ability to enhance animal growth and reduce fat synthesis and are frequently added to animal feed as growth promoters. However, this practice can lead to the presence of *β*-agonist residues in animal products (1). The consumption of such products with high levels of RAC residues can have serious health consequences, including acute poisoning and symptoms such as limb muscle fibrillation, arrhythmia, and hypertension (2–4). Yager (5) administered 1 mg/kg ractopamine to greyhounds, and the dogs showed symptoms of myocardial injury, myocardial necrosis, fibrosis and arterial dysplasia after administration. Adrieli Sachett’s research (6) found that exposing zebrafish to ractopamine caused behavioral changes and oxidative stress in zebrafish. In addition, a study by SUN et al. found that ractopamine affects transcriptional changes in genes related to the hypothalamic–pituitary-gonadal (HPG) axis, which may have the potential to disrupt the endocrine system (7).

RAC has garnered worldwide attention due to its potential safety hazards in animal food (8). The Codex Alimentarius Commission (CAC) sets the maximum residual levels (MRL) of RAC in pigs and cattle, specifying 10 μg/kg in muscles, 10 μg/kg in fat, 40 μg/kg in liver, and 90 μg/kg in kidneys. The acceptable daily intake (ADI) is set at 0–1 μg/kg, with Japan also adhering to CAC standards. In the United States, the Food and Drug Administration (FDA) allows the addition of RAC at levels ranging from 8.2 to 24.6 g/ton to improve protein content and increase lean meat percentage in cattle. For cattle, the MRL of RAC is set at 30 μg/kg in muscles and 90 μg/kg in liver, while for pigs, it is 50 μg/kg in muscles and 150 μg/kg in liver. In New Zealand, the MRL of RAC is set at 10 μg/kg in muscles, 10 μg/kg in fat, 40 μg/kg in liver, and 90 μg/kg in kidneys for pigs. Both China and the European Union have issued directives prohibiting the use of *β*-agonist drugs as feed additives for food animal (9). It is evident that drug residue violations pose a global public health concern (10). Traditionally, drug detection methods rely on animal slaughter, which is complex and costly. Hence, there is a need for more suitable methods to accurately estimate the withdrawal time of drugs in animals used for food production.

The physiologically-based pharmacokinetic model (PBPK) is a comprehensive model that simulates the circulation of blood in the systemic circulation system, taking into account the physiology, chemistry, and anatomy of the body. Each compartment in the model represents specific organs or tissues, and drug transport is determined based on principles of substance balance, considering factors such as actual blood flow rate, tissue/blood partition coefficient, and compound properties (11). The PBPK model has been widely recognized as a reliable method for estimating withdrawal time, as it incorporates mechanistic physiological information, such as drug mode of action, organ-specific exposure, and the influence of diseases on drug disposition, into its predictions (12). It holds significant value in evaluating existing drugs objectively, designing new drugs, and guiding rational drug use. For instance, Cho et al. successfully developed and validated a PBPK model for meloxicam pharmacokinetics in individuals with different CYP2C9 genotypes, aiming to optimize dosing and reduce the risk of adverse events associated with meloxicam use (13). Willemin et al. conducted *in vivo* experiments in rats to refine and calibrate a PBPK model for *trans* and *cis*-benzyl permethrin, effectively capturing toxicokinetic profiles of benzyl permethrin isomers and their metabolites (14). Another study by Sharma et al. involved the development of a detailed human PBPK model for DEHP and its primary metabolites, demonstrating the model’s excellent predictive capability through experimental validation (15). These established PBPK models provide predictions of chemical concentrations in blood and urine under various exposure scenarios, facilitating the exploration of different biological monitoring studies for human health risk assessment. Furthermore, Henri et al. developed a pharmacokinetic model for monensin residues in chickens based on flow limitation physiology, and its predictive power was verified by comparing it to an external dataset that described concentration decay after the end of treatment (16). The application of PBPK model in ruminants also has excellent performance. Leavens et al. established a PBPK model for tulathromycin in goats, which was also extrapolated to juvenile goats. This model effectively simulated plasma and injection site concentrations in juvenile goats using parameters estimated from market-age goats, demonstrating its utility for extrapolating between doses, ages, and species (17). Chou’s research developed PBPK models for flunixin, florfenicol, and penicillin G in cattle and swine, laying the groundwork for more comprehensive models (18). Modern PBPK models have proven useful for estimating WDIs (19–23). PBPK models are mechanism-based, cost-effective, and efficient, enabling extrapolations across exposure paradigms and species.

This study aimed to develop and validate a PBPK model for RAC in goats. The objective was to predict the tissue distribution patterns of RAC and provide valuable insights for the safety assessment and early warning monitoring of “second-generation clenbuterol” drugs in domestic goat production and breeding.

## 2 Methods

### 2.1 Chemicals and reagents

RAC (99.8% isotopic purity) and [^2^H_6_]-RAC (internal standard with a purity of 98.5%) obtained from Dr. Ehrenstorfer GmbH (Augsburg, Germany). Automatic SPE Apparatus (Fotector-06C) were purchased form Reeko Instrument (Xiamen, China). HPLC grade methanol and acetonitrile were provided by Merke, Germany.

### 2.2 Experimental design

This study conducted research on animals in accordance with the regulations of the Feed Research Institute, Chinese Academy of Agricultural Sciences, Beijing, China. 27 healthy male Liaoning cashmere goats aged 10 months and weighing 30 ± 5 kg were included. Before the administration test, the goats underwent a one-week acclimation period in the feeding environment with a drug-free diet. Pharmacokinetic tests were performed on 6 goats following a single oral gavage and a single intravenous administration of RAC. This was followed by a residue depletion test in plasma, urine, and various tissues on the remaining 21 goats (including 3 goats in the control group), which were administered continuous gavage for 28 days.

For the pharmacokinetic study, 6 goats were randomly chosen and subjected to a 12-h fasting period before receiving a single oral dose of RAC at 1 mg/kg BW per day. Blood samples were collected from these goats at 5 min, 10 min, 20 min, 30 min, 1 h, 2 h, 4 h, 6 h, 8 h, 12 h, 24 h, 36 h, 48 h, 72 h, 96 h after administration. After a drug withdrawal period of 15 days, the same six goats received an intravenous injection of RAC at the same dose, and blood samples were collected at 1 min, 5 min, 10 min, 20 min, 30 min, 1 h, 2 h, 4 h, 6 h, 8 h, 12 h, 24 h, 36 h, 48 h, 60 h, 72 h, 84 h, 96 h after administration for pharmacokinetic analysis, and urine samples were obtained from four of the goats.

Residual elimination of RAC in goats was based on data previously published by our laboratory (24, 25). The residual elimination parameters of RAC were determined through detection using ultra-high performance liquid chromatography-quadrupole-orbitrap high-resolution mass spectrometry (UPLC-Q-Orbitrap HRMS). Non-compartmental analysis (NCA) of blood and urine, concentration-time data was performed using WinNonlin software (version 5.2.1).

### 2.3 Apparatus and chromatographic conditions

All the samples were tested according to the a previously published method (26).

### 2.4 Calibration curves and assay validation

The validation of the method began with the analysis of blank tissues using the previously described technique, which revealed no detectable RAC residues. The standard deviation (SD) and the relative standard deviation (RSD = SD/mean × 100%) were determined across the full calibration range. Recovery assessments were conducted at four distinct concentration levels for various tissues and biological fluids: 0.5, 5, 50, and 200 μg/kg for liver, kidney, spleen, lung, heart, fat and brain; 0.5, 5, 10, and 100 μg/kg for muscle tissue; 0.5, 5, 20, and 200 μg/L for plasma; and 0.5, 20, 100, and 500 μg/L for urine.

The samples were examined using UPLC-Q-Orbitrap HRMS, and the signal-to-noise (S/N) ratio was documented. The limits of detection (LOD) and quantification (LOQ) for the analyte were established based on the concentrations in plasma, urine, or different tissues that yielded S/N ratios of 3 and 10, respectively.

Graphs depicting the concentration of the substance in tissue samples (Y, in μg/kg or μg/L) against the time elapsed since treatment cessation (T, in days) were generated using nonlinear regression analysis with GraphPad Prism 6.0 software.

### 2.5 Design of circulation flowchart

The experimental model in this study was constructed using AcslX software (Version 3.2, Aegis Technologies Group Inc). A hybrid PBPK (Physiologically Based Pharmacokinetic) model was developed, which consisted of 10 modules representing various tissues and organs: muscle, spleen, lung, plasma, kidney, fat, heart, brain, liver, and the remaining tissue. In this model, muscle, fat, brain, and the remaining tissue served as membrane-rate-limiting modules, while the remaining tissues and organs were considered flow-limiting modules. The entire code for this model is provided in the Supplementary materials.

**Figure 1:**
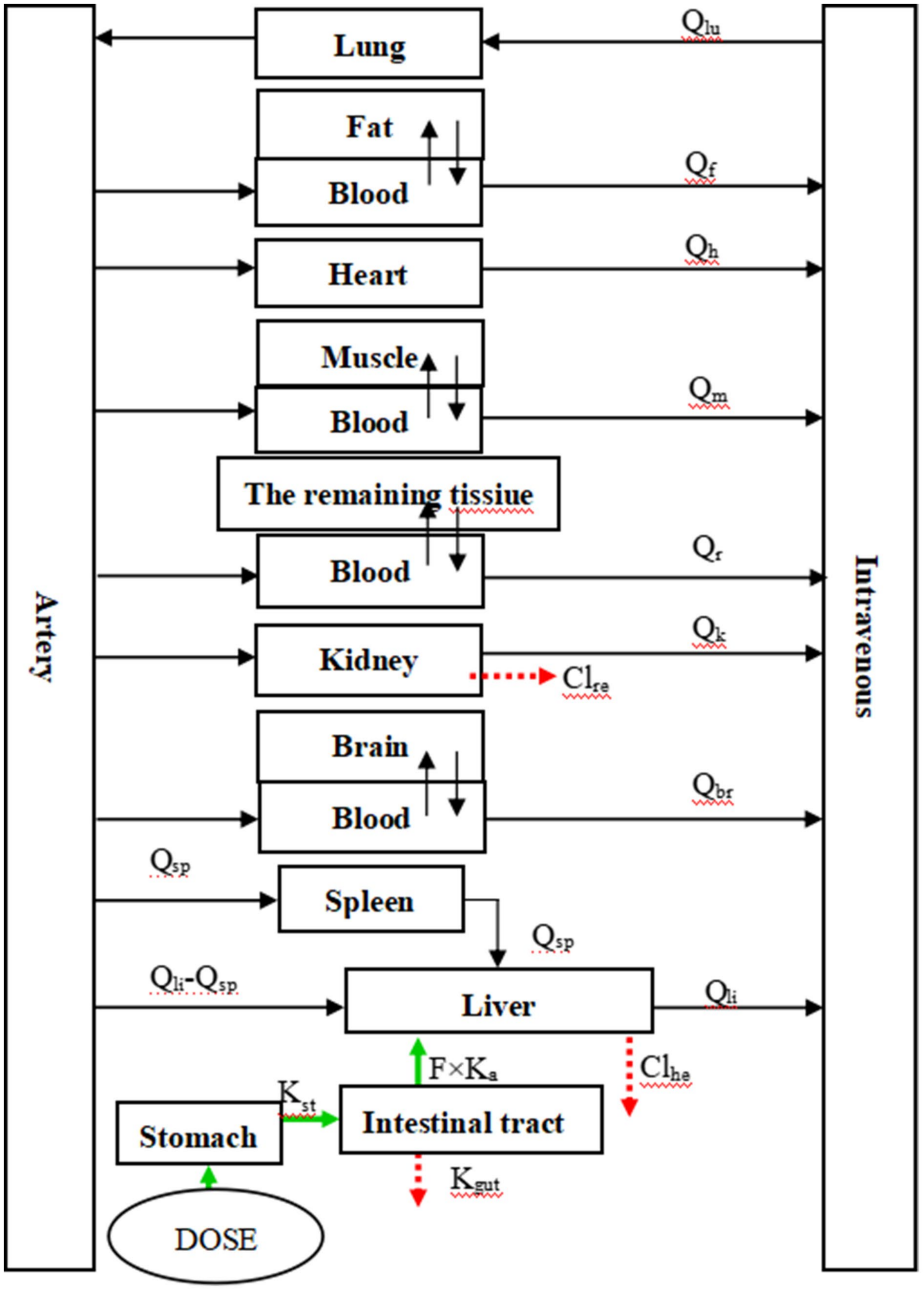
The PBPK model of RAC in goat. Fat, muscle, brain and the remaining tissue in the figure are all membrane rate-limiting modules, while others are blood flow rate-limiting modules.

Figure 1 in the study illustrates the structure of the model. After oral administration, RAC was directly injected into the stomach (simplified as a single gastric compartment). It then passes through the digestive tract and is absorbed by the intestine into the bloodstream, where it is distributed to different tissues and organs. The drug undergoes metabolism in the liver and excretion in the kidneys, with the liver serving as the primary site of metabolism and the kidneys facilitating drug elimination in urine. The rate of gastric emptying is represented by K_st_, while K_a_ represents the absorption rate constant. The unabsorbed fraction is excreted in feces, with K_gut_ used as the rate constant. The bioavailability of RAC, F, is calculated as K_a_/(K_a_ + K_gut_). The study assumes linear elimination of RAC in the liver and kidneys, with Cl_he_ representing the clearance rate in the liver and Cl_re_, indicating the clearance rate in the kidneys (27).

### 2.6 Mass balance equation

Table 1 shows the differential equations that describe the change in drug concentration (or mass) over time in each module, based on the model depicted in Figure 1.

**Table 1:** Mass balance equation of drug concentration change.

Item	Differential equation
Gastric contents	$\frac{dA_{gac}}{dt}=\text{Dose}-K_{st}\times A_{gac}$
Intestinal contents	$\frac{dA_{inc}}{dt}=K_{st}\times A_{gac}-\left(K_{\text{gut}}+F\times K_{a}\right)\times A_{inc}$
Liver	$V_{B}\times \frac{dC_{li}}{dt}=F\times K_{a}\times A_{inc}+\left(Q_{li}-Q_{sp}\right)\times C_{\text{ab}}+Q_{sp}\times \frac{C_{sp}}{P_{sp}}-Q_{li}\times \frac{C_{li}}{P_{li}}-Cl_{\text{he}}\times P_{\text{free}}\times \frac{C_{li}}{P_{li}}$
Spleen	$V_{sp}\times \frac{dC_{sp}}{dt}=Q_{sp}+\left(C_{\text{ab}}-\frac{C_{sp}}{P_{sp}}\right)$
Kidney	$V_{ki}\times \frac{dC_{ki}}{dt}=Q_{ki}+\left(C_{\text{ab}}-\frac{C_{ki}}{P_{ki}}\right)-Cl_{re}\times P_{\text{free}}\times \frac{C_{ki}}{P_{ki}}$
Muscle	$\frac{dC_{mu-\text{blood}}}{dt}\times V_{mu-\text{blood}}=Q_{mu}\times \left(C_{ap}-C_{mu-\text{blood}}\right)+P_{\text{amu}}\times \frac{C_{mu-\text{tissue}}}{P_{mu}}-P_{\text{amu}}\times C_{mu-\text{blood}}$ $\frac{dC_{mu-\text{tissue}}}{dt}\times V_{mu-\text{tissue}}=-P_{\text{amu}}\times \frac{C_{mu-\text{tissue}}}{P_{mu}}+P_{\text{amu}}\times C_{mu-\text{blood}}$
Fat	$\frac{dC_{fa-\text{blood}}}{dt}\times V_{fa-\text{blood}}=Q_{fa}\times \left(C_{ap}-C_{fa-\text{blood}}\right)+P_{afa}\times \frac{C_{fa-\text{tissue}}}{P_{fa}}-P_{afa}\times C_{fa-\text{blood}}$ $\frac{dC_{fa-\text{tissue}}}{dt}\times V_{fa-\text{tissue}}=-P_{afa}\times \frac{C_{fa-\text{tissue}}}{P_{fa}}+P_{afa}\times C_{fa-\text{blood}}$
Heart	$V_{h}\times \frac{dC_{h}}{dt}=Q_{h}\times \left(C_{\text{ab}}-\frac{C_{h}}{P_{h}}\right)$
Lungs	$V_{lu}\times \frac{dC_{lu}}{dt}=Q_{lu}\times \left(C_{vb}-\frac{C_{lu}}{P_{lu}}\right)$
Brain	$\frac{dC_{br-\text{blood}}}{dt}\times V_{br-\text{blood}}=Q_{br}\times \left(C_{br}-C_{fa-\text{blood}}\right)+P_{abr}\times \frac{C_{br-\text{tissue}}}{P_{br}}-P_{abr}\times C_{br-\text{blood}}$ $\frac{dC_{br-\text{tissue}}}{dt}\times V_{br-\text{tissue}}=-P_{abr}\times \frac{C_{br-\text{tissue}}}{P_{mu}}+P_{abr}\times C_{br-\text{blood}}$
The remaining tissue	$\frac{dC_{re-\text{blood}}}{dt}\times V_{re-\text{blood}}=Q_{re}\times \left(C_{re}-C_{re-\text{blood}}\right)+P_{\text{are}}\times \frac{C_{re-\text{tissue}}}{P_{re}}-P_{\text{are}}\times C_{re-\text{blood}}$ $\frac{dC_{re-\text{tissue}}}{dt}\times V_{re-\text{tissue}}=-P_{\text{are}}\times \frac{C_{re-\text{tissue}}}{P_{re}}+P_{\text{are}}\times C_{re-\text{blood}}$
Arterial blood	$V_{\text{ab}}\times \frac{dC_{\text{ab}}}{dt}=Q_{lu}\times \left(\frac{C_{lu}}{P_{lu}}-C_{\text{ab}}\right)$
Venoud blood	$\begin{array}{l} V_{vb}\times \frac{dC_{vb}}{dt}=Q_{mu}\times C_{mu-\text{blood}}+Q_{li}\times \frac{C_{li}}{P_{li}}+Q_{ki}\times \frac{C_{ki}}{P_{ki}}+Q_{re}\times C_{re-\text{blood}}\\ +Q_{h}\times \frac{C_{h}}{P_{h}}+Q_{fa}\times C_{fa-\text{blood}}+Q_{br}\times C_{br-\text{blood}}-Q_{lu}\times C_{vb} \end{array}$

$\frac{dA}{dt}$: the rate of change of drug mass (unit: μg) with time (h) in the organ; Dose: the dose per oral dose, in μg, calculated by multiplying the dose per unit of body weight by the animal’s body weight; Kst: the rate of gastric emptying (h-1); Ka: the absorption rate constant of the drug after oral administration (h-1); F: bioavailability of the drug after oral administration (%); K_gut_: the rate constant (h-1) of the excretion of unabsorbed drugs from the intestinal tract after oral administration; $\frac{dC}{dt}$: the rate of change of drug concentration (C, unit: μg/kg) in the organ with time (h); V: the mass of the organ (kg); Q: blood flow in the organ (L/h); C: drug concentration in the organ (μg/L, the injection of μg/L is equivalent to μg/kg, the same as below); Clhe: the liver clearance rate of the drug (L/h); Clre: renal clearance of the drug (L/h); P: the drug distribution coefficient in organ tissue-plasma (no unit dimension, the same below); Pfree: the free fraction of RAC which numerically equals to 100% minus the plasma protein binding rate (Pbind) of RAC.

In this model, the muscle is divided into two components: muscle fiber cells and the outer fluid of the muscle cells, which represent the blood component of the muscle. A hypothetical membrane is assumed to exist between these two parts. The differential equations for the muscle module involve two variables: C_mu-blood_, which represents the drug concentration in the blood part of the muscle, and C_mu-tissue_, which represents the drug concentration in the muscle tissue. The distribution of drugs between these two parts occurs via osmosis, with Pamu representing the permeability coefficient. The integ function is used to integrate the two differential equations of muscle in Table 1 by acslXtreme software. Consequently, Equations 1 and 2 were derived to calculate the Rac mass of distinct muscle compartments.


(1) $$A_{mu-\text{blood}}=\text{integ}\left(\frac{dC_{mu-\text{blood}}}{dt}*Vmu-\text{blood},0.0\right)$$

(2) $$A_{mu-\text{tissue}}=\text{integ}\left(\frac{dC_{mu-\text{tissue}}}{dt}*Vmu-\text{tissue},0.0\right)$$

Therefore, drug concentration in the whole muscle can be expressed as (A_mu-blood_ + A_mu-tissue_)/V_mu_. Between V_mu-blood_ and V_mu-tissue_ can be expressed by the fraction of blood in the tissue. Furthermore, the distribution of Rac within fat, the brain, and the remaining tissues adheres to the same membrane rate-limiting mechanism as that observed in muscle. Consequently, the quantity of Rac in these tissues can likewise be determined employing this integral method.

### 2.7 Model parameters

The blood flow data for each tissue were obtained through literature references (17, 28), and these values are provided in Table 2. To determine the tissue-organ weight ratios, three healthy male Cashmere goats (10 months old, weighing 30 ± 5 kg) were weighed and then sacrificed. The weights of the heart, liver, lung, kidney, muscle, fat, blood, and other relevant samples are shown in Table 3. In the PBPK model, the tissue-plasma partition coefficient (P) is an important parameter that determines the distribution of drugs in various tissues (29). Since some of these parameters cannot be directly obtained from experiments or literature, it is necessary to employ an optimization model. The AcslX software (Version 3.2, Aegis Technologies Group Inc) includes an optimization module called OptStatModule, which can be utilized for this purpose. The parameters to be optimized in this study include the tissue-plasma partition coefficient, liver clearance rate (Cl_he_), renal clearance rate (Cl_re_), absorption rate constant (K_a_), gastric emptying rate (K_st_), and liver tissue uptake rate constant (K_li_). These parameters are listed in Table 4.

**Table 2:** Tissue blood flow as a percentage of cardiac output.

Organ	Blood flow rate (%)	Organ	Blood flow rate (%)
Liver	48.32	Fat	8.50
Kidney	17.05	Heart	4.98
Muscle	14.00	The remaining tissue	7.15

The whole blood output of the goat was 6.9 L/h/kg; the whole blood output was equal to the blood flow rate in the lungs, and the average weight of the goat used in this project was 30 kg. Blood flow in other tissues equals 100% minus the sum of values in the kidney, liver, muscle, fat, heart, spleen, and brain.

**Table 3:** Organ weights of goat as a percentage of body weight.

Organ	Weight (%)	Organ	Weight (%)
Liver	1.29	Fat	2.74
Kidney	0.31	Brain	0.32
Muscle	35.27	Spleen	0.28
Lungs	0.78	Heart	0.35
The remaining tissue	58.66		

The average weight of the goat was 30 kg; The percentage of other tissues in body weight was obtained by subtracting the sum of liver, kidney, muscle, lung, fat, brain, spleen and heart from 100%, and the specific value was 58.66%.

**Table 4:** Model fitting parameter limits and final values.

Parameter	Unit	Initial value	Final value	Standard deviation
C_lhe_	L/h/kg	0.0633	0.0624	0.000001
C_lre_	L/h/kg	0.0001	0.0001	0.000000
P_pmu_	1	0.0271	0.0271	0.000000
P_pfa_	1	0.0052	0.0054	0.000000
P_pre_	1	0.0000	0.0021	0.000000
P_pbr_	1	0.0064	0.0068	0.000000
K_st_	h^−1^	0.0900	0.0910	0.000001
K_a_	h^−1^	0.98	0.9861	0.000012
K_int_	h^−1^	0.9000	0.9016	0.000011
P_mu_	1	1.0000	1.0686	0.000013
P_fa_	1	0.8	0.7526	0.000009
P_br_	1	1.0000	0.6896	0.000013
P_li_	1	2.3000	2.5584	0.000034
P_ki_	1	1.7000	1.7734	0.000028
P_sp_	1	1.0000	1.0047	0.000012
P_he_	1	1.5	1.3839	0.000023
P_lu_	1	1.4000	1.5333	0.000019
P_re_	1	8.8300	9.0888	0.000244

### 2.8 Validation of the model

To validate the validity of the model, a comparison can be made between the observed concentrations of RAC in each tissue and the predicted concentrations simulated by the model. One way to assess the simulation effect is by performing a linear regression analysis. In the regression analysis, the observed concentrations will serve as the dependent variable, while the predicted concentrations will be the independent variable. By plotting these values on a scatter plot, the slope and intercept of the regression line can be determined. A slope close to 1 and an intercept close to 0 indicate a better simulation effect, as it suggests that the predicted concentrations closely match the observed concentrations. The linear regression analysis provides a quantitative measure of the accuracy and agreement between the model predictions and the observed data.

### 2.9 Sensitivity analysis

Indeed, sensitivity analysis plays a crucial role in the development and application of PBPK models. It helps in identifying the key parameters that have a significant impact on the model predictions. Sensitivity analysis involves assessing the sensitivity of the model to various factors, including physiological, anatomical, and compound-specific parameters, as well as other variables such as body weight, body clearance rate, absorption rate constant, bioavailability, and hematocrit.

Local sensitivity analysis is commonly performed using experimental study samples. It involves observing the changes in model outputs over time by perturbing the parameters of interest. In this case, if the normalized sensitivity coefficient (NSC) reaches a minimum absolute value of 0.25 during the postexposure period, it signifies that the parameter has a significant influence on the drug concentration.

The sensitivity coefficients (SC) are calculated as the relative change in the model output (f(x)) with a relative change in the parameter value (x). These SC values are then normalized to the PBPK parameters, resulting in the NSC. The NSC indicates whether there is a positive or negative correlation between the parameter and the drug concentration. A higher absolute value of NSC suggests a stronger sensitivity of the drug concentration to that specific parameter. The SC and NSC values are calculated from Equations 3 and 4, respectively.


(3) $$SC=\left[f\left(x+\Delta x\right)-f\left(x\right)\right]/\Delta x$$

(4) $$NSC=SC*x/f\left(x\right)$$

### 2.10 Calculate the traceability period

When determining the withdrawal time (WT) for RAC in edible tissues, the maximum residual levels (MRL) established by the Codex Alimentarius Commission (CAC) are taken into consideration. The MRLs for RAC in different tissues are set as follows: 10 μg/kg in muscle, 10 μg/kg in fat, 40 μg/kg in liver, and 90 μg/kg in kidney. To calculate the withdrawal time, the method of Monte Carlo analysis (MCA) can be utilized. This involves performing 500 sampling simulations, representing the disposition process of RAC in 500 individual animals, for all sensitive parameters. These simulations generate 500 sets of concentration-time data for each edible tissue. In the next step, the concentration-time data for RAC in each tissue is compared with the respective MRL. The withdrawal time is then determined as the time at which the residual RAC concentration in each tissue falls below the MRL, taking into account the 95th percentile population with 95% certainty. Calculating the withdrawal time based on this approach ensures that the residual RAC concentration in edible tissues is below the MRL set by the CAC, providing a certain level of safety and compliance.

## 3 Results and discussion

The PBPK model is a holistic conceptual framework that integrates the body’s tissues based on the blood circulatory system, accounting for both the physiological and anatomical features of the animal and the pharmacokinetic properties (ADME) of the drug. Given the complexity of drug disposition, constructing a PBPK model that includes all tissues and ADME processes is nearly impossible. Therefore, simplifying the model by making scientific assumptions and excluding minor factors is necessary. Even with some information omitted, a satisfactory model can still be achieved (30, 31). In this experiment, the researchers considered the four stomachs (rumen, reticulum stomach, flap stomach, abomasum) of goats as a virtual chamber, which is a common approach in PBPK modeling for ruminants (21, 32–34). To develop the PBPK model, they gathered a significant amount of literature to obtain the physiological and anatomical parameters specific to goats. Pharmacokinetics and residual elimination tests were conducted to determine the rate constants for RAC absorption and elimination in goats. The researchers analyzed the variations in these parameters. Considering the structural characteristics of RAC, the research paper initially employed a rate-limiting PBPK model to study the drug’s treatment characteristics. However, they observed that certain tissues could not be adequately simulated using rate-limiting transport, necessitating the use of membrane rate-limiting transport for treatment assessment. By making preliminary modifications to the model, the researchers discovered that the brain, muscle, fat and the remaining tissue exhibited membrane rate-limiting characteristics, while others corresponded to the blood-flow rate-limiting class. This hybrid modeling approach aligns with a study conducted by Cortright regarding the treatment of muscle, fat, and brain, where membrane rate-limiting modules were employed. This trend may be attributed to the fat content present in these specific tissues in goats. These findings highlight the importance of tailoring the PBPK model to accurately represent the unique characteristics of the drug and the specific animal species under investigation. By considering the rate-limiting transport and tissue composition, researchers can develop more refined and accurate models for studying the behavior of RAC in goats (35).

In this model, RAC was rapidly delivered into the stomach following oral administration. During the process of gastric emptying, RAC is transported from the stomach to the intestine along with the chyme and undergoes absorption. Any unabsorbed portion of RAC was excreted through excrement. Once absorbed, RAC was distributed to various tissues and organs in the goat’s body via the bloodstream. In the liver, RAC was metabolized, while in the kidneys, it was excreted through urine. To validate the model’s effectiveness, simulated values were compared with observed values. Plasma, liver, and kidney drug concentrations were assessed to test the model’s sensitivity. The R-squared (R^2^) values were found to be low for both muscle and heart tissues, with predicted values surpassing observed values. This discrepancy can be attributed to RAC’s role as a *β*-adrenoceptor agonist, capable of binding to adrenergic receptors in both the heart and skeletal muscles (36). Although the model considered the rate of RAC binding to blood adrenergic receptors and utilized enzymatic hydrolysis to enhance extraction efficiency, it could not guarantee 100% extraction of bound RAC. Consequently, the measured values were lower than expected. It’s worth noting that the “muscle” module in this PBPK model specifically represents the biceps femoris. Previous research has shown lower RAC accumulation in the biceps brachii compared to other muscles, leading to inadvertent overestimation of its content in the muscle module. These findings suggest potential avenues for refining the model by accurately estimating RAC accumulation in specific tissues and accounting for the drug’s binding properties to adrenergic receptors (25).

### 3.1 Validation of method

Detector responses to RAC were shown to be linear within the concentration range of 0.5–500 μg/L or μg/kg, according to equations *Y* = 0.2397x − 0.4342 (*R*^2^ = 0.9994), *Y* = 0.2557x − 0.3024 (*R*^2^ = 0.9999), *Y* = 0.2142–0.1628 (*R*^2^ = 0.9995), *Y* = 0.2065x − 0.0186 (*R*^2^ = 0.9999), *Y* = 0.1987x + 0.2048 (*R*^2^ = 0.9988), *Y* = 0.2295x + 0.0847 (*R*^2^ = 0.9998), *Y* = 0.1534x + 0.4038 (*R*^2^ = 0.9999), *Y* = 0.1608x + 1.4126 (*R*^2^ = 0.9998), *Y* = 0.1598x + 0.4327 (*R*^2^ = 0.9999), and *Y* = 0.2453 − 0.3873 (*R*^2^ = 0.9996) in heart, liver, spleen, lung, kidney, fat, brain, plasma, urine, and muscle, respectively. The limit of quantification (LOQ) and the limits of detection (LOD) were 0.50 μg/kg or 0.50 μg/L and 0.15 μg/kg or 0.15 μg/L, respectively, to indicate the effectiveness and reliability of the method (24, 25).

### 3.2 Residue depletion study

**Figure 2:**
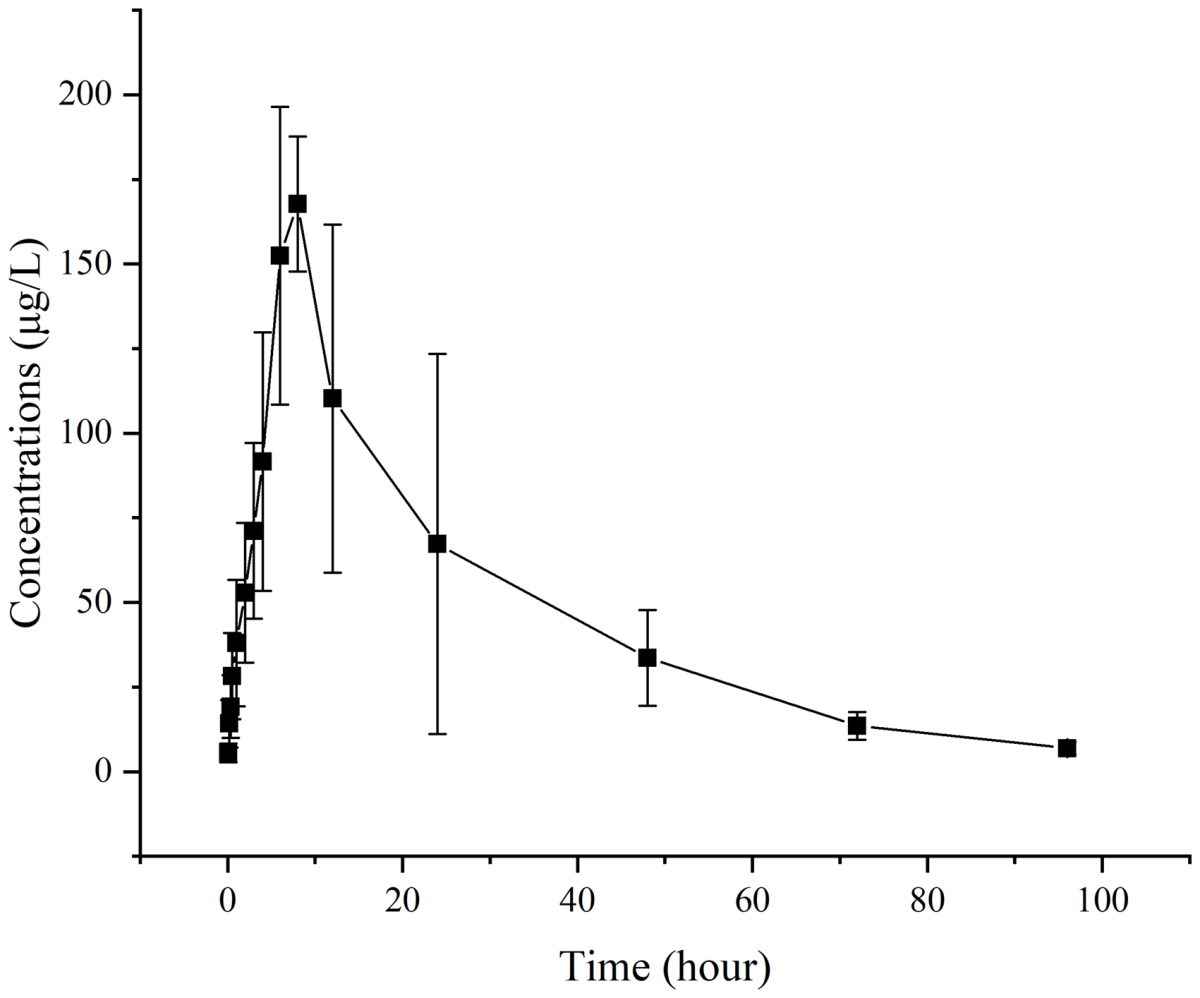
Concentrations (μg/L) of RAC in six goats’ plasma after a single oral administration at 1 mg/kg BW (Specific data are presented in Supplementary materials).

**Figure 3:**
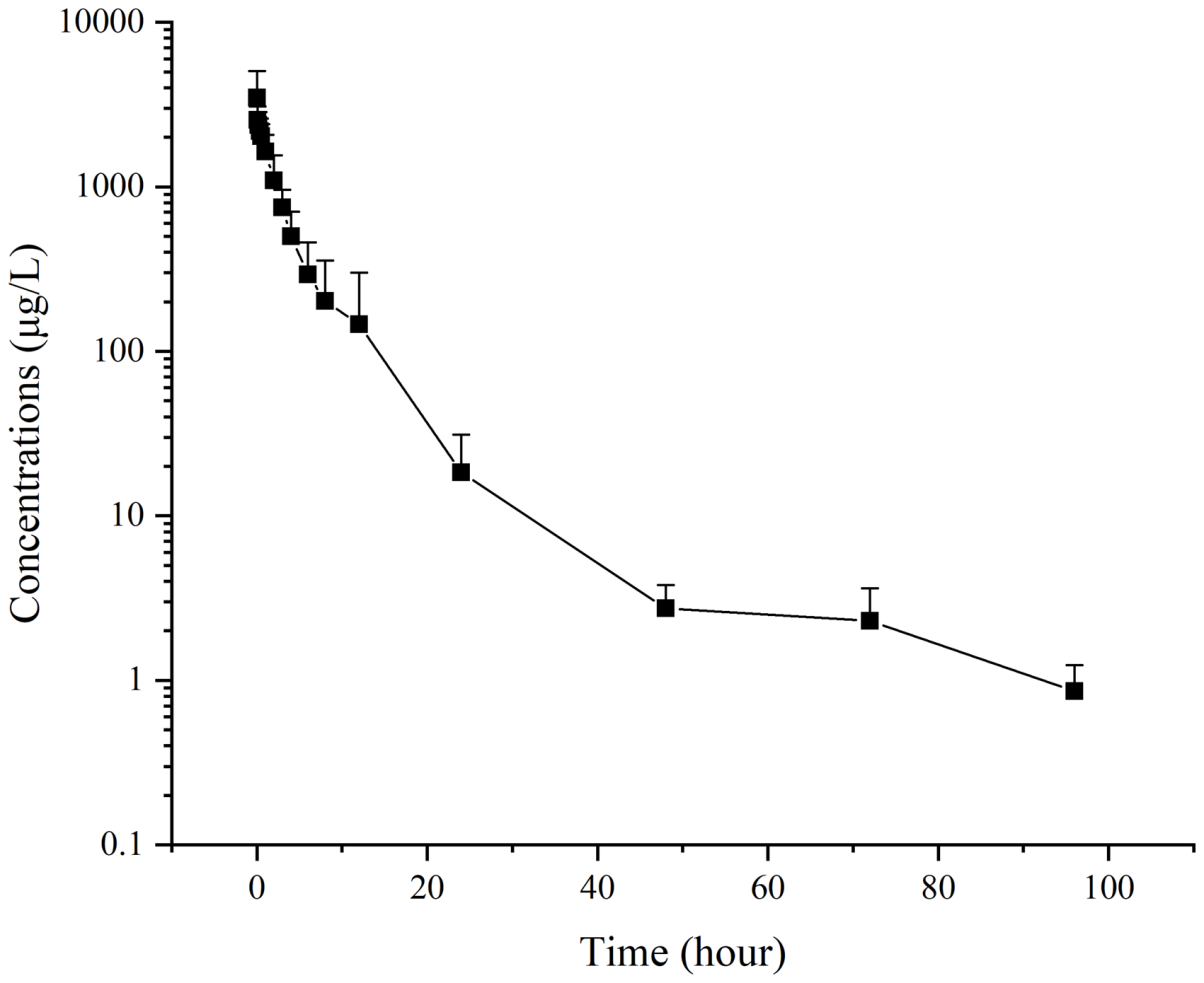
Concentrations (μg/L) of RAC in six goats’ plasma after a single intravenous injection at 1 mg/kg BW (Specific data are presented in Supplementary materials).

**Figure 4:**
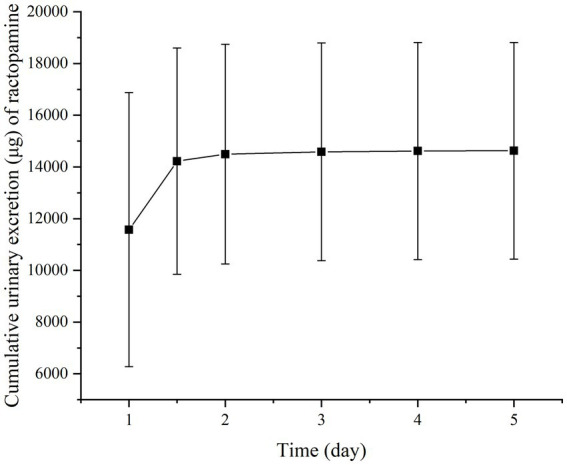
Cumulative urinary excretion (μg) of ractopamine in four goats after a single intravenous injection at 1 mg/kg BW. (Specific data are presented in Supplementary materials).

Figures 2, 3 present the residual RAC levels in the plasma of six goats after a single oral and intravenous administration of RAC at 1 mg/kg BW, respectively. In Figure 4, cumulative urinary excretion (μg) of ractopamine of four goats are shown. In Figures 2, 3, RAC was detected in the plasma just 1 min after intravenous administration, whereas, for oral administration, RAC was first detected in goats 5 min after treatment. Furthermore, the change in RAC concentration in plasma differed between the two administration methods. Following injection, the RAC concentration in plasma peaked at the second minute with an average concentration of approximately 3490.25 μg/L. Subsequently, the residual RAC in plasma gradually decreased and reached less than 1 μg/L after approximately 96 h. On the other hand, for oral administration, the RAC concentration reached its peak at the eighth hour, with an average concentration of approximately 167.74 μg/L. The disparity in results between the two administration methods may be attributed to the faster entry of RAC into the bloodstream in the injection group, leading to a rapid increase in plasma RAC concentration. Conversely, when RAC is administered orally, it needs to be absorbed into the liver through the gastric mucosa before entering the bloodstream, resulting in a longer time to reach peak concentration. As depicted in Figure 4, a sharp increase in excreted RAC in the urine of the four goats was observed on the first day after a single intravenous injection. Subsequently, a gradual decrease in urinary RAC excretion, and after 4–5 days of administration, the concentration of RAC in the urine became too low to be detected. RAC cumulative excretion remained basically unchanged.

Based on the provided data, non-compartmental analysis (NCA) of the plasma and urine concentration-time data was conducted using WinNonlin software (version 5.2.1) to determine the pharmacokinetic parameters of RAC (presented in Table 5).

**Table 5:** Pharmacokinetics parameters of RAC in goat.

	Intravenous injection	Oral administration
T_1/2λZ_ (h)	11.47 ± 2.99	22.29 ± 5.41
C_0_ (μg/L)	2684.49 ± 576.68	
AUC (h·μg/L)	8449.43 ± 3446.82	4636.91 ± 1657.47
AUMC (h^2^·μg/L)	48709.44 ± 26389.81	144753.8 ± 45843.37
Vz (L/kg)	2.39 ± 1.34	
Cl (L/h/kg)	0.14 ± 0.06	
MRT (h)	5.49 ± 0.99	31.64 ± 3.78
Vss (L/kg)	0.72 ± 0.22	
F (%)	54.88	

### 3.3 Validation of the model

**Figure 5:**
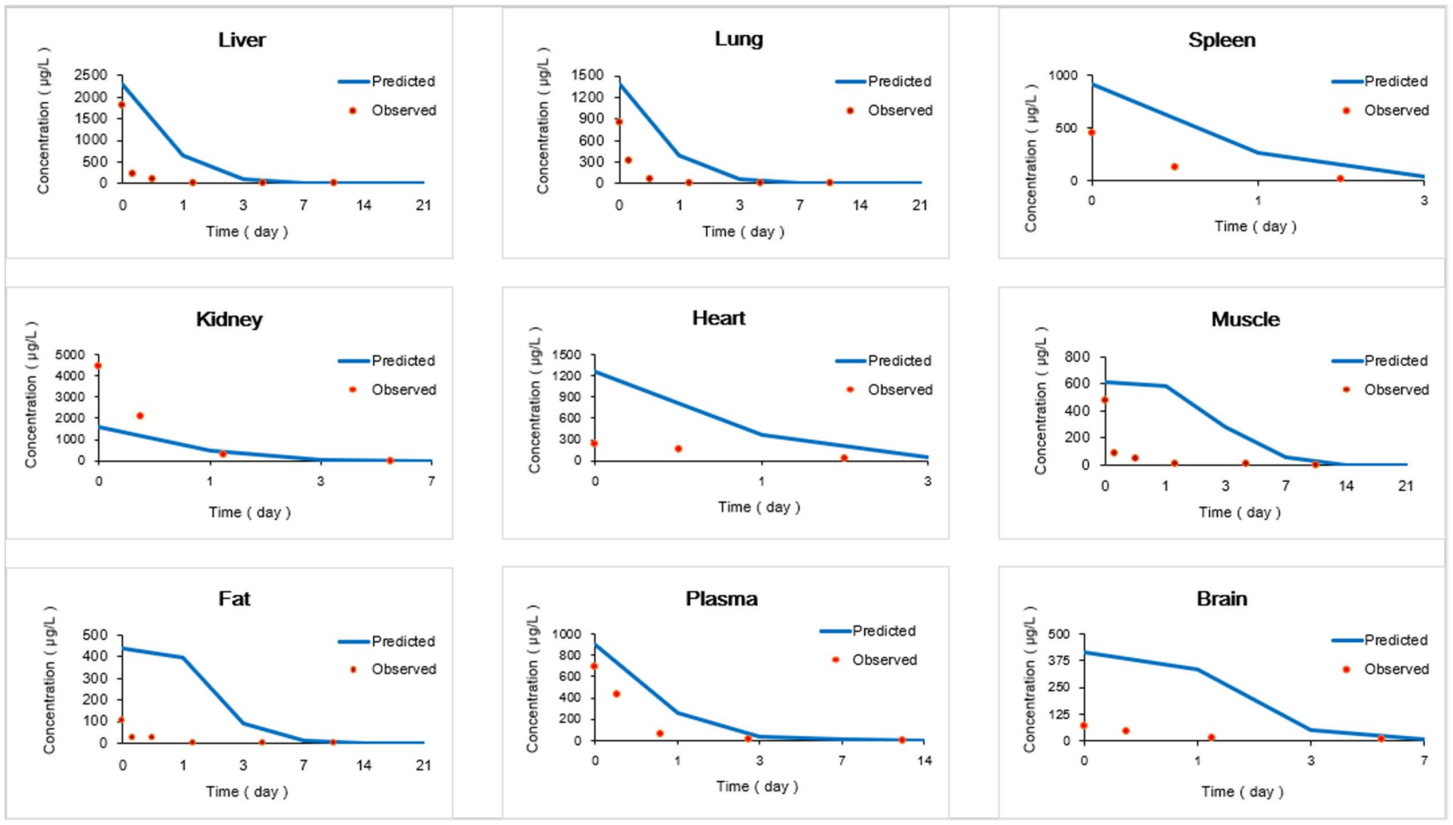
Comparisons of observed and predicted RAC concentrations (μg/kg). (Specific data are presented in Supplementary materials).

**Figure 6:**
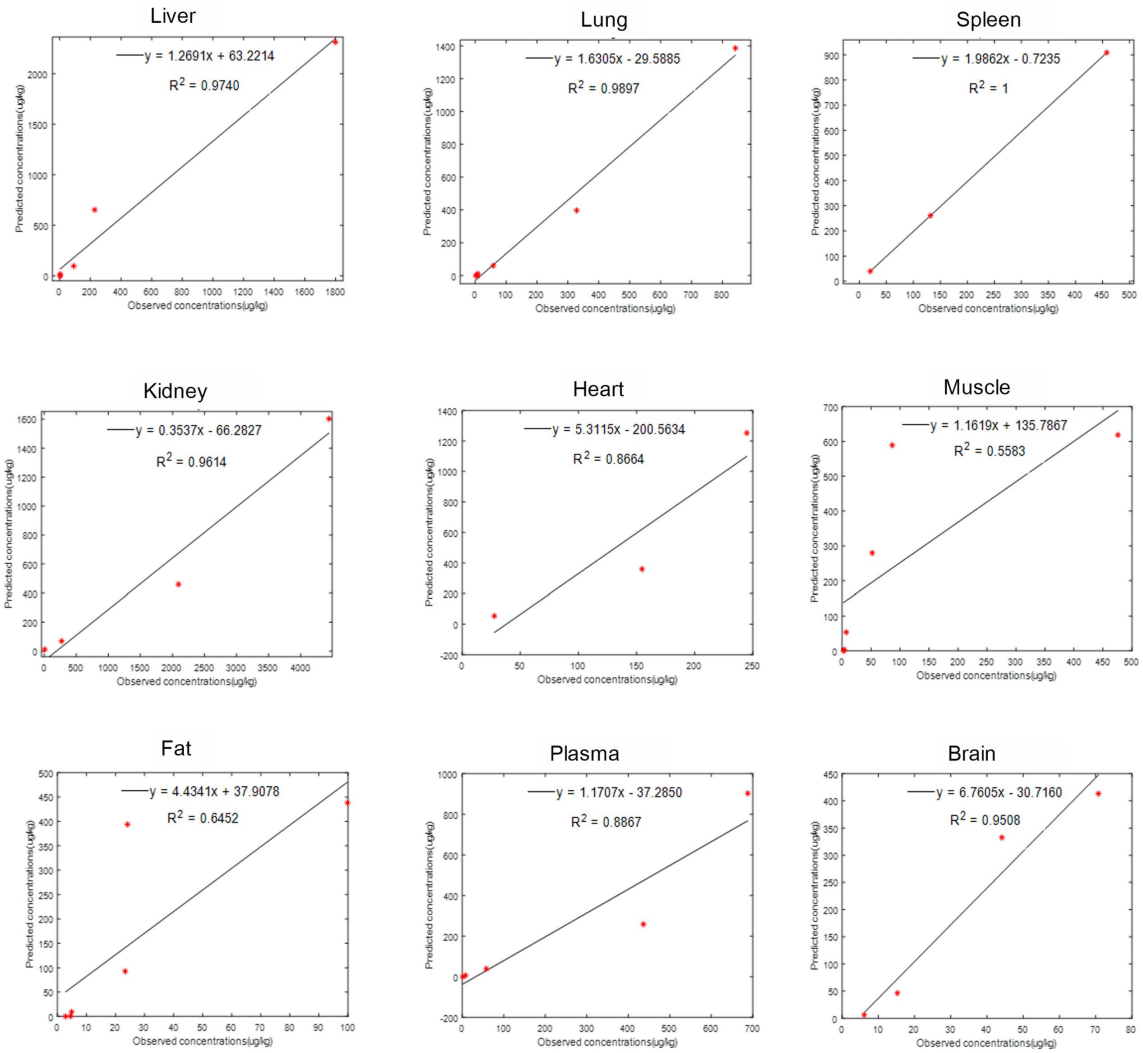
The linear regression analysis between observed (points) and predicted (curves) RAC concentrations in goat tissues.

**Figure 7:**
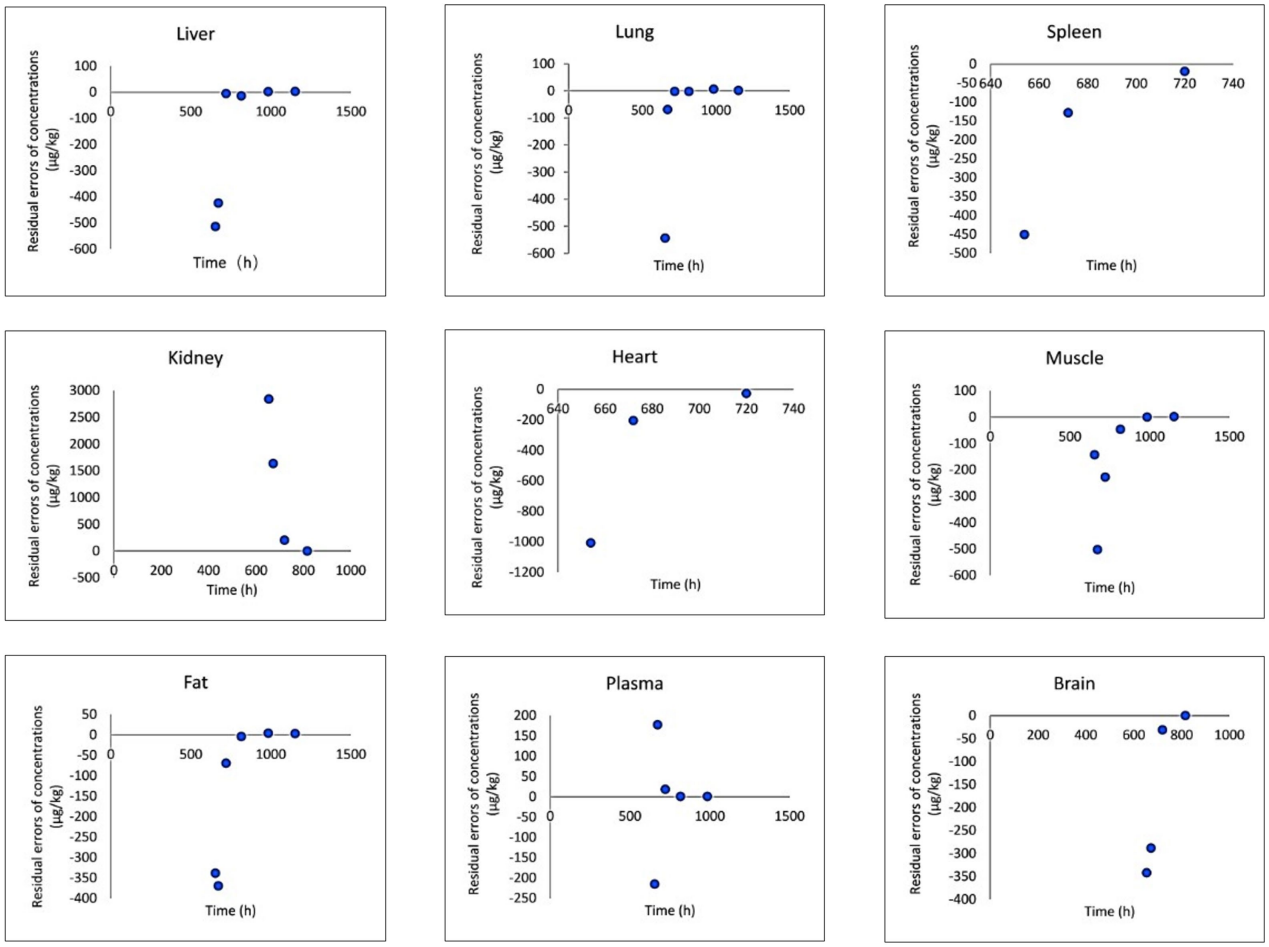
Result of residual analysis between observed and predicted RAC concentrations in goat tissues.

The provided data includes a comparison of observed and predicted RAC concentrations in nine organs of goats exposed to RAC through oral gavage at a dosage of 1.0 mg/kg BW for 28 consecutive days (presented in Figure 5). The highest observed residual concentration of RAC was found in the kidney, with higher concentrations also observed in the liver, lung, and spleen. The variation in residual RAC amounts in different tissues may be attributed to the varying distribution of *β*-receptors within these tissues. According to Elisinga (37), the density of β-receptors in the heart, lung, kidney, liver, and spleen is higher compared to that in the intestine, fat, bone, and cerebellum. It is worth noting that the RAC concentration in the plasma decreased to 1 μg/L after 21 days of drug withdrawal, indicating a relatively slow metabolism. Regression analysis and residual error analysis between the predicted and observed values are shown in Figures 6, 7, respectively. The linear regression analysis in Figure 6 demonstrates that the model exhibits excellent predictive ability and good coverage for most tissues. The R-squared (R^2^) values for the liver, lung, spleen, kidney, heart, muscle, fat, plasma, and brain were 0.9740, 0.9897, 1.0000, 0.9614, 0.8664, 0.5583, 0.6452, 0.8867, and 0.9508, respectively. The *R*^2^ values for the liver, lung, kidney, and brain were all above 0.9. Based on the combined analysis of linear regression (Figure 6) and residual analysis (Figure 7), it was observed that the measured and predicted values of RAC in the plasma fell along the regression line, and the residual values were evenly distributed on both sides of the *x*-axis. Therefore, the model effectively predicted the concentration level of RAC in the goat plasma. Regarding liver and lung tissue, the residual values are closely aligned with the coordinate axis, except for the first two points. The linear analysis R^2^ values were 0.9740, 0.9897, indicating that the model successfully simulated the residual levels of RAC in the these tissues 1 days after drug withdrawal. For the heart, fat, and muscle, the RAC contents could be accurately simulated 3 day, 7 days, and 14 days after drug withdrawal, respectively. Similarly, for kidney, the RAC contents were well-predicted 7 days after drug withdrawal. Additionally, the predicted values of all the models exhibited the same trend of concentration change. The use of PBPK (Physiologically-Based Pharmacokinetic) models to address drug residues in animals is growing in research. PBPK models can replace certain animal experiments and significantly improve experimental efficiency. Previous studies have successfully employed PBPK models to predict drug exposure and enhance inter-species extrapolation of dosing regimens or withdrawal period calculations (38). For example, Leavens et al. constructed a goat PBPK model for tobramycin (17), a macrolide antibiotic, and achieved a successful simulation of its pharmacokinetics. In our experiment, oral administration of RAC was used. If the injection method had been employed instead, the model parameters would have been further optimized to provide more accurate and sensitive predictions for the residual period.

### 3.4 Sensitivity analysis

Sensitivity analysis was used to identify the parameters in the model that had the greatest impact on RAC concentration in the plasma, liver, and kidney. Parameters such as P, absorption and disposal of ractopamine in goat tissues were optimized using the measured data in the OptStatModule of AcslX (VerSion 3.2, Aegis Technologies Group Inc) software. Based on the results presented in Table 6, the parameter Q_clu_ was found to be the most sensitive, exhibiting the highest influence on RAC concentration in the plasma, liver, and kidney. Furthermore, Q_clu_ displayed a negative correlation with drug concentration. The absolute values of the NSC (Normalized Sensitivity Coefficient) for parameters Q_cli_, Q_cki_, Q_cmu_, Q_cfa_, Cl_he_, Q_che_, P_re_, K_int_, pcv, and Q_cbr_ were all at least 0.75, indicating significant effects on RAC concentration with a negative correlation. These parameters had a considerable impact on the RAC concentration. Other sensitive parameters such as BW (body weight), P_pre_, Ka (absorption rate constant), V_cmu_ (volume of the central compartment), and P_bind_ also had a notable influence on drug concentration, with NSC values above 0.38. These parameters demonstrated a positive correlation with RAC concentration. In terms of specific organs, the parameter P_li_ had a significant effect on RAC concentration in the liver, while P_ki_ had a significant effect on RAC concentration in the kidney. These parameters were positively correlated with drug concentration and played a crucial role in determining RAC concentrations in their respective organs.

**Table 6:** Results of NSCs to RAC concentration in plasma, liver and kidney.

Coefficient	Value	Coefficient	Value	Coefficient	Value
cvp:bw	3.817996	cli:bw	3.804836	cki:bw	3.817928
cvp:ppre	1.979063	cli:ppre	1.979065	cki:ppre	1.979064
cvp:ka	0.940319	cli:pli	1.000059	cki:pki	1.000010
cvp:vcmu	0.634308	cli:ka	0.940319	cki:ka	0.940319
cvp:pbind	0.384382	cli:vcmu	0.634310	cki:vcmu	0.634309
cvp:qcbr	−0.753019	cli:pbind	0.394398	cki:pbind	0.384434
cvp:pcv	−0.772010	cli:qcbr	−0.753020	cki:qcbr	−0.753019
cvp:kint	−0.940325	cli:pcv	−0.792681	cki:pcv	−0.772117
cvp:pre	−0.949182	cli:kint	−0.940325	cki:kint	−0.940325
cvp:qche	−1.875017	cli:pre	−0.949184	cki:pre	−0.949183
cvp:clhe	−1.934316	cli:qche	−1.875019	cki:qche	−1.875018
cvp:qcfa	−3.200331	cli:clhe	−1.984771	cki:clhe	−1.934316
cvp:qcmu	−5.271677	cli:qcfa	−3.200334	cki:qcfa	−3.200333
cvp:qcki	−6.419485	cli:qcmu	−5.271683	cki:qcmu	−5.271681
cvp:qcli	−18.242790	cli:qcki	−6.419492	cki:qcki	−6.419227
cvp:qclu	−80.828470	cli:qcli	−18.192390	cki:qcli	−18.242800
		cli:qclu	−80.828470	cki:qclu	−80.828470

### 3.5 Calculate the traceability period

After performing 500 Monte Carlo simulations, the simulated concentrations of RAC in each tissue of 500 virtual individuals were compared with the corresponding Maximum Residue Limit (MRL) in each tissue. This analysis aimed to determine the time at which the drug concentration after the last administration dropped below the MRL value in each tissue. This allowed us to obtain the Withdrawal Time (WT) of RAC in goat tissues.

**Figure 8:**
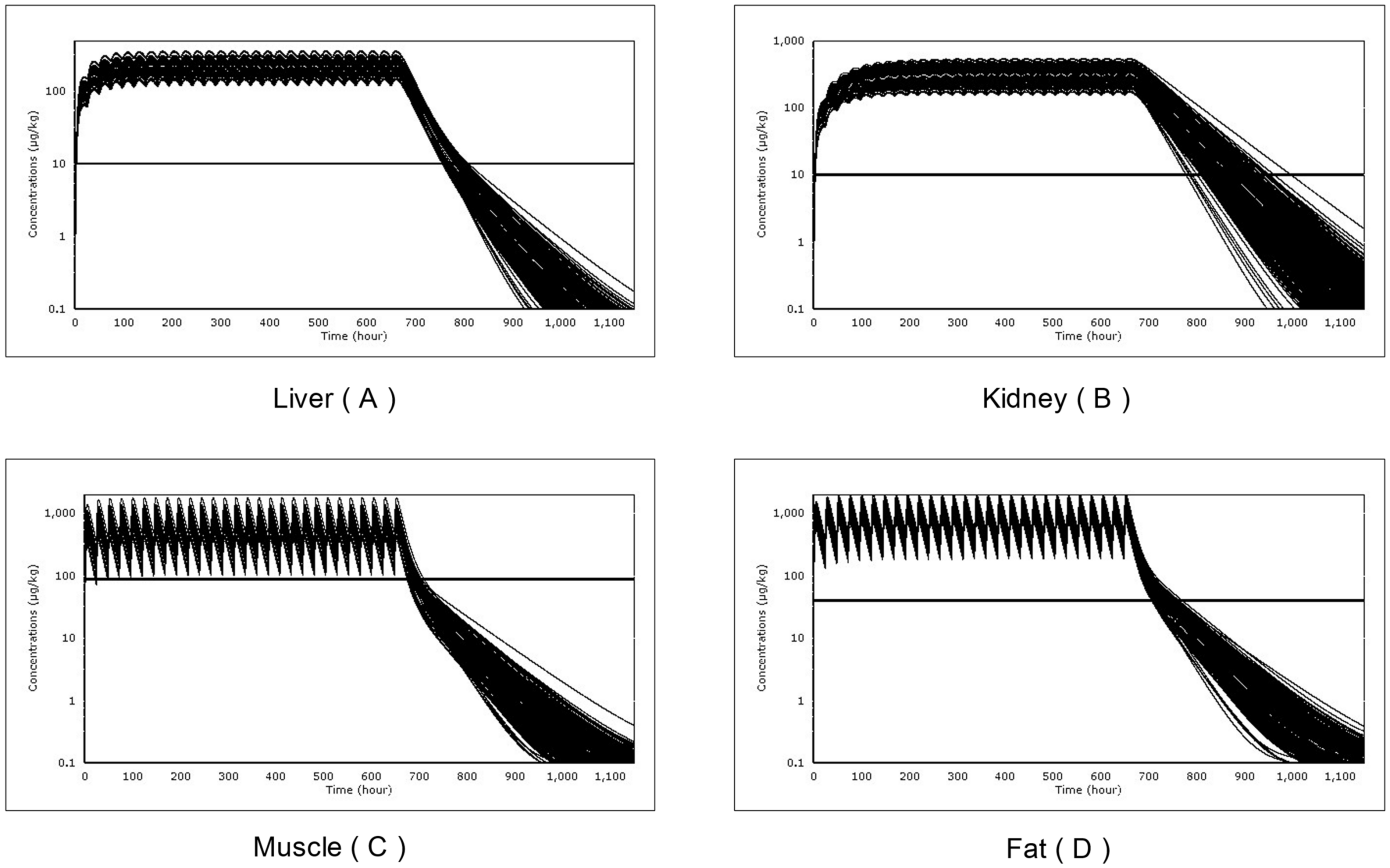
The Withdrawal time (WT) of times of RAC in goat tissues after a single Monte Carlo analysis of 500 simulations: liver **(A)**, kidney **(B)**, muscle **(C)** and fat **(D)**.

The residual concentrations of RAC in the four target tissues (muscle, liver, kidney, and fat) were compared with their respective MRLs, as shown in Figure 8. By programming in acslXtreme software, the earliest time at which the RAC concentration in each tissue fell below the corresponding MRL after the last dose was automatically identified. Additionally, statistical analysis with a 95% confidence limit was conducted to calculate the WT of RAC residue in the four target tissues based on the dosing scheme used in the study. These results are presented in Table 7.

**Table 7:** Distributions of the Withdrawal time of RAC in goats.

Organ	WT (hour)	WT (day)
Muscle	38	2
Liver	138	6
Kidney	310	13
Fat	100	5

After 28 days of continuous oral gavage at a dose of 1.0 mg/kg BW, the WT of RAC in muscle, liver, kidney, and fat tissues was determined to be 2 days, 6 days, 13 days, and 5 days, respectively. The kidney had the longest traceability period among the four tissues. This information allows us to predict the period during which RAC residues can be detected in these edible tissues.

The Monte Carlo method was utilized to establish a realistic feed exposure scenario, based on the oral gavage model, in order to simulate the depletion of RAC in edible tissues after drug exposure through feed. This approach is consistent with the methodology employed in previous PBPK models (30, 39).

## Data Availability

The original contributions presented in the study are included in the article/Supplementary material, further inquiries can be directed to the corresponding authors.
