# Corrigendum: Development and application of a physiologically-based pharmacokinetic model for ractopamine in goats

**DOI:** 10.3389/fvets.2024.1523431

**Published:** 2024-11-21

**Authors:** Jing Ai, Yunfeng Gao, Fan Yang, Zhen Zhao, Jin Dong, Jing Wang, Shiyi Fu, Ying Ma, Xu Gu

**Affiliations:** ^1^Institute of Feed Research of Chinese Academy of Agricultural Sciences, Beijing, China; ^2^Heilongjiang Technical Appraisal Station of Agricultural Products, Veterinary Pharmaceuticals and Feed, Harbin, China; ^3^College of Animal Science and Technology, Henan University of Science and Technology, Luoyang, China; ^4^Beijing Nutrient Source Research Institute Co., Ltd., Beijing, China; ^5^ZiBo Government Service Center, Zibo, Shandong, China; ^6^Jiangxi Agricultural Technology Extension Center, Nanchang, China

**Keywords:** ractopamine, goats, physiologically-based pharmacokinetic model, physiological parameters, residues

In the published article, there was an error in the Funding statement. A fund, “Agricultural Science and Technology Innovation Program of the Feed Research Institute of the Chinese Academy of Agricultural Sciences (CAAS-IFR-ZDRW202402)” was omitted. The correct Funding statement appears below.

