# Supplementary material for: Development and application of a physiologically-based pharmacokinetic model for ractopamine in goats
Source: Front Vet Sci. 2024 Oct 2;11:1399043. doi: 10.3389/fvets.2024.1399043 (PMC11479929; doi:10.3389/fvets.2024.1399043)
Supplement: Supplementary file 1 [file Table_1.docx]

Development and application of a physiologically-based pharmacokinetic model for ractopamine in goats

Ai Jing^1†^，Gao Yunfeng^2^ , Yang Fan^3^, Zhao Zhen^4^, Jin Dong^5^ ,Wang Jing^1^, Fu Shiyi^6^, Ma Ying^1^*, Gu Xu^1^*

^1^Institute of Feed Research of Chinese Academy of Agricultural Sciences, Beijing 100081, China

^2^Heilongjiang Technical Appraisal Station of Agricultural Products, Veterinary Pharmaceuticals and Feed, Harbin, 150036, China

^3^College of Animal Science and Technology, Henan University of Science and Technology, Luoyang 471023, China

^4^Beijing Nutrient Source Research Institute Co., LTD., Beijing 100071, China

^5^ZiBo Government Service Center, 119 Xisi Road, Zhangdian District, Zibo City, Shandong Province, China

^6^Jiangxi Agricultural Technology Extension Center, Nanchang 330046, China

Supplementary Materials

1 Supplementary Tables……………………………………………………………………………..2

**Table S1.** Concentrations (μg/L) of RAC in goat plasma after a single oral administration................................................................................................2

**Table S2.** Concentrations (μg/L) of RAC in goat plasma after a single intravenous injection........................................................................................................3

**Table S3.** Cumulative (μg) urinary excretion of RAC in four goats after a single intravenous injection........................................................................................4

**Table S4.** Comparisons of observed and predicted RAC concentrations (μg/kg).............5

2 PBPK model code written in acslXtreme software…………………………………...6

**1 Supplementary Tables**

**Table S1.** Concentrations of RAC in six goats’ plasma after a single oral administration (μg/L)

| Blood collection time | Number | | | | | | Mean±SD |  |
| --- | --- | --- | --- | --- | --- | --- | --- | --- |
|  |  |  |  |  |  |  |  |  |
|  | 1 | 2 | 3 | 4 | 5 | 6 |  |  |
| 5 min | ND | 9.13 | 7.01 | 2.02 | 4.12 | 5.23 | 5.5±2.72 |  |
| 10 min | 5.8 | 24.81 | 17.9 | 7.8 | 12.81 | 15.9 | 14.17±6.97 |  |
| 20 min | 7.84 | 31.59 | 25.8 | 9.84 | 17.59 | 22.8 | 19.24±9.27 |  |
| 30 min | 13.22 | 44.56 | 39.3 | 15.22 | 24.56 | 32.3 | 28.19±12.76 |  |
| 1 h | 22.02 | 72.39 | 28.7 | 24.02 | 42.39 | 38.7 | 38.04±18.65 |  |
| 2 h | 30.8 | 85.11 | 56.7 | 32.8 | 65.11 | 46.7 | 52.87±20.65 |  |
| 3 h | 53.83 | 117.35 | 57.3 | 53.83 | 87.35 | 57.3 | 71.16±26 |  |
| 4 h | 68.73 | 168.31 | 76.3 | 70.73 | 89.4 | 76.3 | 91.63±38.25 |  |
| 6 h | 105.13 | 226.09 | 113.2 | 173.29 | 148.23 | 149 | 152.49±43.97 |  |
| 8 h | 155.92 | 180.6 | 142.4 | 158.15 | 171.19 | 198.2 | 167.74±19.89 |  |
| 12 h | 65.11 | 188.16 | 156.6 | 59.75 | 93.94 | 97.96 | 110.25±51.43 |  |
| 24 h | 29.68 | 56.49 | 180.6 | 47.48 | 44.72 | 44.64 | 67.27±56.19 |  |
| 48 h | 19.25 | 54.23 | 48.2 | 24.79 | 25.04 | 30.28 | 33.63±14.19 |  |
| 72 h | 10.34 | 17.74 | 15.7 | 9.14 | 10.29 | 17.98 | 13.53±4.05 |  |
| 96 h | 4.84 | 7 | 7.8 | 5.01 | 6.78 | 9.87 | 6.88±1.87 |  |

Note: RAC was administered at 1 mg/kg BW.

ND stands for not detected.

**Table S2.** Concentrations of RAC in six goats’ plasma after a single intravenous injection (μg/L)

| Blood collection time | Number | | | | | | | Mean±SD |
| --- | --- | --- | --- | --- | --- | --- | --- | --- |
|  | 1 | 2 | 3 | 4 | 5 | 6 |  | |
| 1 min | 3515.46 | 2319.05 | 3009.97 | 1864.1 | 5524.86 | 4709.68 | 3485.53±1571.09 | |
| 5 min | 1731.32 | 2301.72 | 2927.2 | 1749.18 | 2951.63 | 2832.25 | 2552.4±521.1 | |
| 10 min | 2694.32 | 2297.35 | 2765.35 | 1571.78 | 2519.74 | 2705.86 | 2372.02±483.2 | |
| 20 min | 2060.63 | 2057.54 | 2687.89 | 1549.03 | 2333.57 | 2262.79 | 2178.16±418.78 | |
| 30 min | 1835.44 | 2016.1 | 2466.48 | 1436.13 | 2064 | 2190 | 2034.54±377.51 | |
| 1 h | 1514.61 | 1322.26 | 1947.61 | 1123.12 | 1637.39 | 2170.58 | 1640.19±431.36 | |
| 2 h | 929.73 | 531.49 | 1506.38 | 666.57 | 1340.94 | 1427.81 | 1094.64±458.69 | |
| 3 h | 643.52 | 790.49 | 935.4 | 411.13 | 718.15 | 897.71 | 750.58±208.37 | |
| 4 h | 360.8 | 333.6 | 673.67 | 228.43 | 621.47 | 650.48 | 501.53±205.54 | |
| 6 h | 148.17 | 148.92 | 375.23 | 120.39 | 296.96 | 526.15 | 293.53±167.1 | |
| 8 h | 158.5 | 68.94 | 368.03 | 63.01 | 363.13 | 150.42 | 202.71±152.65 | |
| 12 h | 16.33 | 41 | 402.6 | 34.93 | 177.8 | 72.71 | 145.81±154.59 | |
| 24 h | 39.24 | 12.89 | 37.65 | 3.75 | 22.94 | 14.86 | 18.42±12.73 | |
| 48 h | ND | 2.65 | 1.49 | 2.01 | 4.09 | 3.45 | 2.74±1.05 | |
| 72 h | ND | 2.62 | 1.15 | 0.7 | 3.21 | 3.82 | 2.3±1.33 | |
| 96 h | ND | 0.87 | 0.41 | 0.57 | 1.27 | 1.2 | 0.86±0.38 | |

Note: RAC was administered at 1 mg/kg BW.

ND stands for not detected.

**Table S3.** Cumulative urinary excretion of ractopamine in four goats after a single intravenous injection (μg)

| Urine collection  Time | Number | | | | Mean±SD |
| --- | --- | --- | --- | --- | --- |
|  | 1 | 2 | 3 | 4 |  |
| 1 d | 7880.88 | 7393.20 | 18839.20 | 12186.36 | 11574.91±5300.20 |
| 1.5 d | 11290.20 | 12109.22 | 20719.90 | 12777.74 | 14224.27±4372.94 |
| 2 d | 11543.32 | 12822.38 | 20804.17 | 12807.20 | 14494.27±4249.09 |
| 3 d | 11670.83 | 12994.73 | 20836.62 | 12827.71 | 14582.47±4210.79 |
| 4 d | 11752.72 | 13023.86 | 20854.17 | 12835.91 | 14616.65±4195.90 |
| 5 d | 11787.26 | 13023.86 | 20854.17 | 12837.13 | 14625.61±4187.90 |

Note: RAC was administered at 1 mg/kg BW.

ND stands for not detected；V stands for the volume of urine.

**Table S4.** Comparisons of observed and predicted RAC concentrations (μg/kg)

| Organ  Time | Liver | | Lung | | Spleen | |
| --- | --- | --- | --- | --- | --- | --- |
|  | Observed | Predicted | Observed | Predicted | Observed | Predicted |
| 0 d | 1798.95 | 2312.803 | 841.7 | 1385.197 | 457.88 | 908.836 |
| 1 d | 228.47 | 652.755 | 328.35 | 396.901 | 131.8 | 260.638 |
| 3 d | 91.65 | 96.453 | 58.83 | 60.656 | 20.23 | 39.773 |
| 7d | 3.31 | 16.954 | 8.58 | 10.695 | ND | ND |
| 14d | 2.93 | 1.094 | 6.96 | 0.690 | ND | ND |
| 21d | 2.90 | 0.228 | 1.71 | 0.144 | ND | ND |
| Organ  Time | Kidney | | Heart | | Muscle | |
|  | Observed | Predicted | Observed | Predicted | Observed | Predicted |
| 0 d | 4440.39 | 1603.102 | 244.84 | 1252.932 | 475.77 | 618.557 |
| 1 d | 2093.56 | 459.603 | 154.7 | 359.521 | 86.4 | 589.059 |
| 3 d | 271.69 | 70.169 | 27.64 | 54.813 | 52.03 | 279.688 |
| 7d | 8.24 | 12.372 | ND | ND | 7.18 | 53.443 |
| 14d | ND | ND | ND | ND | 3.825 | 2.994 |
| 21d | ND | ND | ND | ND | 2.435 | 0.238 |
| Organ  Time | Fat | | Plasma | | Brain | |
|  | Observed | Predicted | Observed | Predicted | Observed | Predicted |
| 0 d | 99.83 | 438.323 | 687.87 | 903.179 | 70.75 | 413.092 |
| 1 d | 24.09 | 393.913 | 436.48 | 258.750 | 44.11 | 332.457 |
| 3 d | 23.33 | 92.643 | 57.99 | 39.553 | 15.27 | 46.406 |
| 7d | 4.9 | 9.207 | 8.14 | 6.975 | 6.10 | 6.158 |
| 14d | 4.55 | 0.517 | 1.38 | 0.450 | ND | ND |
| 21d | 2.8 | 0.081 | ND | ND | ND | ND |

Note: ND stands for not detected

2 PBPK model code written in acslXtreme software

1
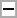
 PROGRAM


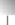

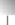


2

1.
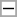
 INITIAL
2. constant pcv=**0.29**, QAR=**6.9**, bw=**30**
3. Vcre=**1**-(Vcli+Vcki+Vclu+Vcmu+Vcfa+Vche+Vcab+Vcvb+Vcsp+Vcbr)
4. Qcre=**1**-(Qcli+Qcki+Qcmu+Qcfa+Qche+Qcbr)
5. **!! Volume (V); Blood flow (Q); partition coefficient(P);
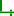
Permeability constants; Volume of blood in some tissue**

**! liver**

**! kidney**

| 8 |  | constant | Vcli=**0.0129**, | Qcli=**0.4832**, | Pli=**2.558408** |  |
| --- | --- | --- | --- | --- | --- | --- |
| 9 |  | constant | Vcki=**0.0031**, | Qcki=**0.1705**, | Pki=**1.77341** |  |
|  |  |  |  |  |  |  |
| 10 |  | constant | Vclu=**0.0078**, | Qclu=**1**, | Plu=**1.533326** |  |
|  |  |  |  |  |  |  |
| 11 |  | constant | Vcmu=**0.3527**, | Qcmu=**0.14**, | Pmu=**1.068578**, |  |

**! lung**

PPmu=**0.02712495**, Vfmu=**0.01 !**

#
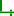
muscle

1. constant Vcfa=**0.0274**, Qcfa=**0.085**, Pfa=**0.7526104**,
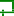


PPfa=**0.00537883**, Vffa=**0.005 !**

#
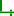
fat

1. constant Vche=**0.0035**, Qche=**0.0498**, Phe=**1.383948**
2. constant Vcab=**0.0188
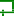
**

# ! heart

# ! artery blood

1. constant Vcvb=**0.0376
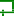
**

# ! venous blood

1. constant Vcsp=**0.0022**, Qcsp=**0.04**, Psp=**1.004675
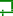
**

# ! spleen

1. constant Vcbr=**0.005**, Qcbr=**0.02**, Pbr=**0.6896452**,
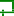


PPbr=**0.006772557**, Vfbr=**0.01 !
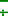
**

# brain
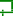


**! brain**

1. constant Pre=**9.088835**,
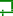


PPre=**0.002063587**, Vfre=**0.02 !**

#
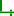
rest of body

1. **!! volume of each tissue or organ**
2. Vli=bw*Vcli; Vki=Vcki*bw; Vlu=Vclu*bw; Vmu=Vcmu*bw; Vfa=Vcfa*
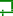

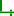
bw; Vhe=Vche*bw; Vab=Vcab*bw; Vvb=Vcvb*bw; Vre=Vcre*bw
3. Vsp=Vcsp*bw; Vbr=bw*Vcbr
4. Vap=Vab*(**1**-pcv) **! volume of artery plasma, not artery blood**
5. Vvp=Vvb*(**1**-pcv) **! volume of venous plasma, not venous blood**

24

25 VBLANCE=bw-(Vli+Vki+Vlu+Vmu+Vfa+Vhe+Vab+Vvb+Vre+Vsp+Vbr) **!
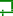
**
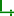
**Check whether the sum of each tissue or organ is equal to the
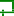
**
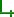
**dodyweight!**

26

# !! volume of blood in some tissue, volume of some tissue except
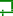

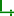
the blood inside it. Please note that both of these parameters
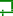

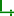
only apply to membrane limited tissues or organs.

1. Vmub=Vmu*Vfmu; Vmut=Vmu-Vmub **! muscle**
2. Vfab=Vfa*Vffa; Vfat=Vfa-Vfab **! fat**
3. Vreb=Vre*Vfre; Vret=Vre-Vreb **! rest**
4. Vbrb=Vbr*Vfbr; Vbrt=Vbr-Vbrb **! brain**

# !! Q ! Blood or plasma flow

1. QTOT=QAR*bw****0.74***(**1**-pcv) **! cardiac output flows, it is for
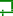
**
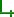
**plasma, not for blood!**
2. Qli=QTOT*Qcli; Qki=QTOT*Qcki; Qlu=QTOT*Qclu **! liver,
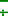
**
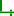
**kidney, and lung, respectively!**


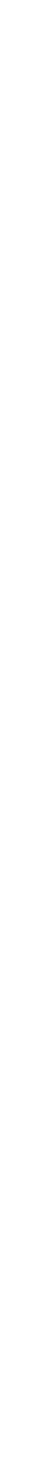


1. Qmu=QTOT*Qcmu; Qfa=QTOT*Qcfa; Qhe=QTOT*Qche **! muscle
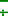
**
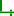
**, fat, and heart, respectively!**
2. Qre=QTOT*Qcre; Qbr=QTOT*Qcbr; Qsp=QTOT*Qcsp **! the
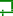
**
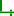
**rest of body, spleen, and brain, respectively!**

37

1. QBLANCE=QTOT-(Qli+Qki+Qmu+Qfa+Qhe+Qre+Qbr) **!!!! Check
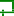
**
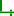
**whether the sum of each plasma flow through tissues is equal to
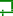
**
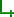
**the cardiac output (plasma)!**

# !! Permeability !! Please note that this parameter only apply
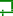

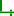
to membrane limited tissues or organs.

1. PAmu=PPmu*Qmu; PAfa=PPfa*Qfa; PAre=PPre*Qre; PAbr=PPbr*
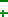

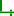
Qbr

# ! ! Kinetic constants

1. **! Oral absorption rate constants**

43 constant kst=**0.09103672**, ka=**0.9861192**, kint=**0.9016421 ! kli=0.5
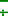
**
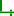
**, kga=0.6**

# ! rates of gastric emptying, absorption, intestinal
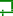

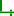
elimination, hepatic elimination, and biliary excretion,
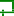

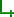
respectively.

1. F=ka/(ka+kint)

# ! The bioavailability of Clenbuterol in goat after oral
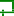

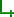
administration.

47

# ! ! multiple oral dose

1. CONSTANT PDOSEpo = **2000 !(ug/kg/day body weight), single
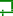
**
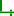
**oral dose**
2. dosepo=PDOSEpo*bw **!ug**
3. CONSTANT tlen = **0.001 ! Length of oral gavage exposure (
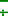
**
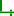
**h/day)**

# CONSTANT tinterval = 24 ! Varied dependent on the exposure
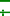

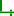
paradigm, in this study the Clenbuterol was administrated once a
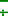

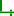
day. So a 24 h was chosen here.

1. CONSTANT Dstart = **0.0 ! Initiation day of oral gavage (
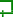
**
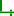
**day). Please note the unite is day!**
2. CONSTANT Dstop = **1 !28 ! Termination day of oral
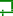
**
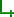
**gavage (day).**
3. CONSTANT MAXT = **1.0 ! maximum comm. interval**
4. CONSTANT CINTC = **0.1 ! Communication interval**
5. CINT = CINTC **! Communication interval**
6. Tsim = TSTOP **! Tstop in hours**
7. DS = Dstart***24 ! Initiation time point of oral gavage (h)**
8. Doff = (Dstop - Dstart)***24 ! Oral gavage duration (h)**
9. TimeOn = Dstart***24 ! Initiation time point of oral gavage
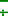
**
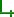
**(h)**
10. TimeOff = Dstop***24**+tlen **! Termination time point of oral
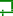
**
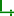
**gavage (h)**

# ! elimination from liver and kidney

64 constant clhe=**0.06243522**, clre=**0.0001090409**, pbind=**0.1651634 !
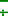
**
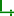
**hepatic clearance (L/h/kg), renal clearance (L/h/kg), and plasma
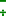
**
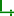
**protein binding ratio, respectively!**

65 CCLhe=clhe*bw; CCLre=clre*bw **! hepatic and renal
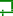
**
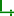
**clearance (L/h)**

66 pfree=**1**-pbind **! Unbound fraction**

67

68

69 END **! INITIAL**

72
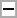
 DYNAMIC


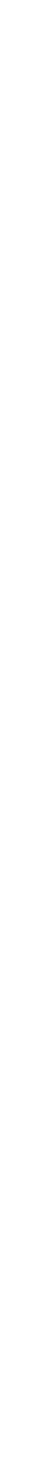

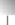

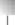


73

1. ALGORITHM IALG = **2**
2. NSTEPS NSTP = **10**
3. MAXTERVAL MAXT = **1.0e9**
4. MINTERVAL MINT = **1.0e**-**9**
5. CINTERVAL CINT = **0.1**

79

80
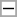
 DERIVATIVE

81

# ! multiple oral administration

1. Exposure = PULSE(DS, Tsim, Doff)*PULSE(**0**,tinterval,tlen)
2. Rdosepo = (dosepo/tlen)*Exposure
3. **! Concentration (c) of ractopamine in each tissue/organ or or mass
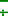
**
   1. **of ractopamine in alimentary canal contents.**
4. **! stomac**
5. rsto=Rdosepo-kst*asto
6. asto=integ(rsto,**0**) **!!**
7. raintogut=kst*asto
8. intogut=integ(raintogut,**0**)

# ! gut

1. rgut=kst*asto-kint*agut-ka*F*agut
2. agut=integ(rgut,**0**) **!!**
3. regut=kint*agut
4. egut=integ(regut,**0**) **!!**

# ! liver

1. rali=(Qli-Qsp)*Cap+Qsp*Csp/Psp-Qli*Cli/Pli+ka*F*agut-reli
2. ali=integ(rali,**0**) **!!**
3. Cli=ali/Vli

10 reli=CCLhe*Cli/Pli*pfree

10 eli=integ(reli,**0**) **!!**

10 **! spleen**

10 rasp=Qsp*(Cap-Csp/Psp)

10 asp=integ(rasp,**0**)

10 Csp=asp/Vsp

10 **! kidney**

10 raki=Qki*(Cap-Cki/Pki)-reki

10 aki=integ(raki,**0**) **!!**

10 Cki=aki/Vki

11 reki=CCLre*Cki/Pki*pfree

11 eki=integ(reki,**0**) **!!**

11 **! muscle**

11 ramub=Qmu*(Cap-Cmub)+PAmu*Cmut/Pmu-PAmu*Cmub

11 amub=integ(ramub,**0**)

11 Cmub=amub/Vmub

11 ramut=-PAmu*Cmut/Pmu+PAmu*Cmub

11 amut=integ(ramut,**0**)

11 Cmut=amut/Vmut

11 Cmu=amu/Vmu

12 amu=amub+amut **!!**

12 **! heart**

12 rahe=Qhe*(Cap-Che/Phe)

12 ahe=integ(rahe,**0**)

12 Che=ahe/Vhe

12 **! fat**

12 rafab=Qfa*(Cap-Cfab)+PAfa*Cfat/Pfa-PAfa*Cfab

12 afab=integ(rafab,**0**)

12 Cfab=afab/Vfab

12 rafat=-PAfa*Cfat/Pfa+PAfa*Cfab

13 afat=integ(rafat,**0**)

13 Cfat=afat/Vfat


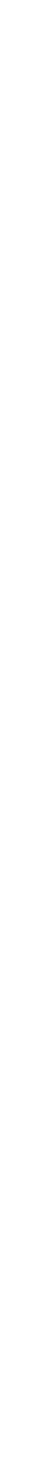


13 Cfa=afa/Vfa

13 afa=afab+afat **!!**

13 **! brain**

13 rabrb=Qbr*(Cap-Cbrb)+PAbr*Cbrt/Pbr-PAbr*Cbrb

13 abrb=integ(rabrb,**0**)

13 Cbrb=abrb/Vbrb

13 rabrt=-PAbr*Cbrt/Pbr+PAbr*Cbrb

13 abrt=integ(rabrt,**0**)

14 Cbrt=abrt/Vbrt

14 Cbr=abr/Vbr

14 abr=abrb+abrt **!!**

14 **! rest**

14 rareb=Qre*(Cap-Creb)+PAre*Cret/Pre-PAre*Creb

14 areb=integ(rareb,**0**)

14 Creb=areb/Vreb

14 raret=-PAre*Cret/Pre+PAre*Creb

14 aret=integ(raret,**0**)

14 Cret=aret/Vret

15 Cre=are/Vre

15 are=areb+aret **!!**

15 **! lung**

15 ralu=Qlu*(Cvp-Clu/Plu)

15 alu=integ(ralu,**0**)

15 Clu=alu/Vlu

# 15 ! arterial plasma

15 raap=Qlu*(Clu/Plu-Cap)

15 aap=integ(raap,**0**)

15 Cap=aap/Vap

# 16 ! venous plasma

16 ravp=Qre*Creb+Qbr*Cbrb+Qfa*Cfab+Qhe*Che/Phe+Qmu*Cmub+Qki*Cki
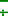

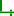
/Pki+Qli*Cli/Pli-Qlu*Cvp

16 avp=integ(ravp,**0**)

16 Cvp=avp/Vvp

# 16 ! mass blance

16 BLANCE=intogut-(agut+abr+asp+ali+aki+amu+afa+ahe+are+alu+aap
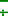

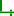
+avp)-(egut+eli+eki)

# 16 !! Check the mass balance.

16 END **! DERIVATIVE**

16

16 CONSTANT TSTOP = **1152.0**

17 TERMT (T .GE. TSTOP,
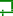


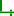
**'checked on communication interval: REACHED TSTOP'**)

17 END **! DYNAMIC**

17 END **! PROGRAM**
